# Learning curve analysis of 100 consecutive robotic liver resections

**DOI:** 10.1007/s00464-025-11551-5

**Published:** 2025-02-27

**Authors:** Mirhasan Rahimli, Andrew A. Gumbs, Aristotelis Perrakis, Sara Al-Madhi, Maximilian Dölling, Frederike Stelter, Eric Lorenz, Mihailo Andric, Mareike Franz, Jörg Arend, Roland S. Croner

**Affiliations:** 1 Department of General, Visceral, Vascular and Transplant Surgery, University Hospital Magdeburg, Leipziger Str. 44, 39120 Magdeburg, Germany; 2 Department of Surgery, Centre Hospitalier Intercommunal de Poissy/Saint-Germain-en-Laye, 10 Rue du Champ Gaillard, 78300 Poissy, France

**Keywords:** Robotic surgery, Liver surgery, Hepatectomy, Learning curve, HCC, CRLM

## Abstract

**Background:**

Robotic liver surgery, as part of minimally invasive liver surgery (MILS), offers advantages like enhanced dexterity and stable camera and instrument control. However, the learning curve — particularly the number of cases required for proficiency — remains underexplored. This study analyzes 100 consecutive robotic liver resections performed by a single surgeon to assess the learning curve and outcomes.

**Patients and methods:**

This retrospective analysis reviewed data from the Magdeburg registry of minimally invasive liver surgery (MD-MILS) from June 2013 to July 2024, to identify surgeons who performed at least 100 robotic liver resections. Operation time and Iwate difficulty score were used as indicators of surgical proficiency and learning progression.

**Results:**

One surgeon fulfilled the inclusion criteria of performing 100 robotic liver resections. Of these, 41 (41%) were major and 59 (59%) were minor liver resections. A significant reduction in average operation time was observed across the series, decreasing from an initial average of 450 min to approximately 300 min by the 100th operation. Similarly, the Iwate difficulty score showed a significant upward trend, reflecting the increasing technical challenges of the procedures. The learning curve was divided into four distinct phases: Competency (Cases 1–20), Proficiency (Cases 21–30), Early Mastery (Cases 31–65), and Full Mastery (Cases 66–100), highlighting the progression in skill acquisition and case complexity. Major complications (21%), such as bile leak, bilioma, and pulmonary embolism, were observed during the study period.

**Conclusion:**

Over 100 robotic liver resections, significant learning curve progression was evident, with marked improvements in operation time and handling of complex cases. The results suggest that approximately 65 cases are needed for a surgeon experienced in open and laparoscopic liver surgery to achieve consistent proficiency in robotic liver resections. These findings underscore the importance of standardized training protocols to support the learning curve in robotic liver surgery.

The benefits of minimally invasive liver surgery (MILS) for the patients is still controversially discussed. Although retrospective data analysis and prospective randomized trials have identified less morbidity and better perioperative outcomes with equal oncological results compared to open procedures, the majority of patients undergoes still open surgery [1–7]. The reasons for this maybe the challenges which are provided during the learning curve of MILS.

The complexity of the liver, resembling a vascular-rich sponge with approximately 100 ml of blood flow per minute per 100 g of liver tissue, presents unique challenges during liver surgery [8]. Even with advanced imaging and intraoperative ultrasound, the variable density of the hepatic parenchyma and hidden vascular structures pose significant difficulties, particularly in cases with fibrosis or cirrhosis [9–11]. These factors contribute to the hesitancy among hepatobiliary surgeons to adopt MILS more broadly.

There is an ongoing discussion about the best training programs and standardization of MILS. Difficulty scores regarding different procedures, fellowship programs, and technical standardizations have been provided to get people started. These helpful tools give surgeons safety and guidance through the learning process. The patient safety must be guaranteed during all steps of the learning curve [12–15].

A recent systematic review has highlighted the importance of standardized approaches to understanding learning curves in minimally invasive liver surgery (MILS). A comprehensive analysis of 60 studies proposed a three-phase learning curve model — competency, proficiency, and mastery — and emphasized the need for clear definitions and standardized reporting in surgical training programs. This model identifies key metrics such as operative time, blood loss, conversion rates, and complications as critical endpoints for assessing learning curves and improving patient safety [16].

Concurrently, advancements in robotic technology have introduced new features that potentially expand the capabilities of MILS, though the utility of robotics in liver surgery is still debated. Some steps, such as parenchymal dissection, continue to present challenges [9, 10, 17].

The learning curve and technical standardization of robotic-assisted MILS are not as extensively explored as those of conventional laparoscopic MILS. After gaining experience with conventional MILS, we became the first group in Germany to implement robotic MILS in 2013 [18, 19]. In this study, we share insights into the learning curve of robotic-assisted MILS, drawing from our experiences and data collected from over 100 robotic liver resections performed by a single surgeon.

## Patients and methods

### Patients

This retrospective analysis reviewed data from the Magdeburg registry of minimally invasive liver surgery (MD-MILS) from June 2013 to July 2024, to identify surgeons who performed at least 100 robotic liver resections. One surgeon met the selection criteria, having performed over 100 robotic liver resections, of which the first 100 consecutive procedures were included in the analysis. The majority of these procedures (80%) were conducted after 2018, reflecting an increased adoption of robotic techniques during this period. Robotic ALPPS procedures were excluded from the study to avoid result distortion, as these surgeries consist of two separate stages. Initially, robotic liver surgery was primarily performed on patients requiring minor resections. With increasing surgical experience, the indications were progressively expanded to include major liver resections starting in 2018. Currently, robotic liver surgery is the standard approach in our center, with nearly all liver resections, including complex techniques such as the ALPPS procedure, performed fully robotically. Exceptions include cases requiring multivisceral resections or vascular reconstructions, where an open approach is preferred for optimal control and outcomes.

### Definitions

The resection of ≥ 3 segments was considered as a major resection [20]. We used the Iwate difficulty scoring system to assess the severity of robotic liver resections. This scoring system was specifically designed for minimally invasive liver surgery and provides a comprehensive evaluation of factors such as tumor location, size, proximity to major vessels, and the extent of liver resection, which were particularly relevant to our study [21].

Postoperative hospital stay was defined as the length of stay (LOS). The final diagnosis, number of intrahepatic lesions, and the size of the largest lesion were determined based on histopathological examination.

Overall morbidity included all postoperative complications, with liver surgery-related complications such as posthepatectomy liver failure, postoperative bleeding, bile leak, bile fistula, bilioma, cholangitis, cholangiosepsis, liver abscess, and portal vein thrombosis categorized using the Clavien-Dindo classification [22].

### Robotic liver surgery

Robotic liver surgery was performed using the da Vinci Si System, and since September 2019, the da Vinci Xi system (Intuitive Surgical Inc., Sunnyvale, CA, USA). Liver parenchyma dissection was carried out using the harmonic scalpel in combination with bipolar forceps or monopolar scissors combined with the Waterjet (ERBEJET® 2, Erbe Elektromedizin GmbH, Tuebingen, Germany) [17].

Right and left hemihepatectomies were conducted as previously published [23, 24]. Since 2018, indocyanine green fluorescence imaging has been utilized during minimally invasive liver surgery [24]. Additionally, the falciform ligament flap sealing technique, published in July 2020, was employed for covering the liver resection surface where anatomical conditions permitted [25].

### Statistical analysis

Patient demographics, perioperative parameters, tumor characteristics, and surgical procedures were analyzed using descriptive and inferential statistics. Continuous variables were summarized as means and standard deviations (SD), while categorical variables were expressed as frequencies and percentages.

To assess the learning curve, operation time and Iwate difficulty score were evaluated across four phases identified by cumulative average analysis. Linear regression models were applied to each phase to determine trends over time, with slope, intercept, R-squared values, and p-values calculated to assess significance.

Comparisons between phases for continuous variables were conducted using the Kruskal–Wallis test, followed by Man Whitney U test for pairwise comparisons when appropriate. The chi-square test was used to assess differences in categorical variables across phases.

P-values less than 0.05 were considered statistically significant. The statistical analyses were conducted using IBM SPSS Statistics for Windows, Version 26 (IBM Corp., Armonk, NY, USA).

## Results

### Baseline parameters

The baseline characteristics of the cohort are summarized in Table 1. The study included 100 patients with a mean age of 62.0 years (SD 13.0) and a mean BMI of 27.5 kg/m^2^ (SD 5.0). The cohort was predominantly male (60%). Most patients had an ASA score of 2 (50%), followed by scores of 3 (42%), 1 (7%), and 4 (1%). A history of previous abdominal surgery was noted in 54 patients (54%).

**Table 1:** Patient demographics, surgical characteristics, and perioperative outcomes of robotic liver resections

		*n* or mean (% or SD)
Total		100 (100)
Sex	Male	60 (60.0)
	Female	40 (40.0)
Age; years		62.0 (13.0)
BMI; kg/m^2^		27.5 (5.0)
ASA	1	7 (7.0)
	2	50 (50.0)
	3	42 (42.0)
	4	1 (1.0)
Iwate difficulty score		7.7 (2.1)
Difficulty level	Low (0–3)	0 (0.0)
	Intermediate (4–6)	35 (35.0)
	Advanced (7–9)	44 (44.0)
	Expert (10–12)	21 (21.0)
Operation time; min		310.6 (114.4)
LOS; days		11.6 (10.5)
IBL; ml		344.0 (315.4)
Intraoperative blood transfusion		12 (12.0)
Overall morbidity		28 (28.0)
Major complication (Clavien-Dindo Grade ≥ 3a)		21 (21.0)
Liver surgery-related morbidity		12 (12.0)
Conversion		9 (9.0)
Previous abdominal surgery		54 (54.0)
Extent of resection	Major	41 (41.0)
	Minor	59 (59.0)
Tumor dignity	Malignant	78 (78.0)
	Benign	22 (22.0)
Size of tumor; cm		55.3 (36.3)
Tumor size groups	< 3 cm	27 (27.0)
	3–5 cm	26 (26.0)
	5.1–10 cm	36 (36.0)
	> 10 cm	11 (11.0)
Number of lesions	1	68 (68.0)
	2	13 (13.0)
	3	7 (7.0)
	> 3	7 (7.0)
R status in malignant cases	R0	74 (94.9)
	R1	4 (5.1)

*ASA* American Society of Anesthesiologists, *BMI* body mass index, *IBL* intraoperative blood loss, *LOS* length of stay, *SD* standard deviation

### Intraoperative features

Key intraoperative parameters are detailed in Table 1. The mean operation time was 310.6 min (SD 114.4), with a mean intraoperative blood loss of 344.0 ml (SD 315.4). Blood transfusions were required in 12 cases (12%). The mean Iwate difficulty score was 7.7 (SD 2.1), and 65% of the procedures were performed at an advanced or expert difficulty level, indicating a substantial portion of complex cases. After successfully completing the first 21 minor resections, the surgeon expanded to major resections starting with the 22nd operation.

A total of 9 conversions were recorded, corresponding to a conversion rate of 9%. The primary reasons for conversion included anesthesiological issues, intraoperative bleeding, and technical challenges.

### Postoperative outcomes

Postoperative outcomes are summarized in Table 1. Overall morbidity occurred in 28 patients (28%), with liver surgery-related complications in 12 cases (12%). Major complications (Clavien-Dindo Grade ≥ 3a) were observed in 21 patients (21%), with bile leakage (5 cases) and bilioma (4 cases) being the most frequent. Other major complications, such as pneumonia, pulmonary embolism, and intra-abdominal fluid collections, were also noted. The mean length of hospital stay was 11.6 days (SD 10.5).

### Oncologic outcomes

Oncologic outcomes are presented in Tables 1 and 2. The majority of indications for robotic liver surgery were malignant lesions (78%), with the remainder being benign (22%). The mean tumor size was 55.3 mm (SD 36.3), with 68% of patients having a single lesion. Almost half of the patients presented with tumors exceeding 5 cm in size. An R0 resection was achieved in 94.9% of malignant cases, with R1 margins in 5.1%.

**Table 2:** Distribution of liver lesion types

		Total *n* (%)
Malignant lesions		78 (78.0)
	Colorectal liver metastases	35 (35.0)
	HCC	24 (24.0)
	CCA	13 (13.0)
	Mixed HCC + CCA	2 (2.0)
	Other liver metastases	4 (4.0)
Benign lesions		22 (22.0)
	FNH	6 (6.0)
	Hepatic adenoma	5 (5.0)
	Liver hemangioma	4 (4.0)
	Liver cyst	2 (2.0)
	Others	5 (5.0)
Total		100 (100.0)

*CCA* cholangiocarcinoma, *FNH* focal nodular hyperplasia, *HCC* hepatocellular carcinoma

Detailed types of liver lesions are listed in Table 2. Among the malignant diagnoses, colorectal liver metastases were the most common (35 cases), followed by hepatocellular carcinoma (HCC, 24 cases) and cholangiocarcinomas (CCA, 13 cases). Benign lesions included focal nodular hyperplasia (FNH, 6 cases), hepatic adenoma (5 cases), and liver hemangioma (4 cases).

### Procedures performed

The types of robotic liver resections are summarized in Table 3. A total of 41 major resections (41.0%) were performed, including right and left hemihepatectomies and extended resections. Minor resections (59.0%) included left lateral liver resections (24 cases) and bisegmentectomies (16 cases). This range of procedures reflects the surgical approach tailored to the specific characteristics of each liver lesion.

**Table 3:** Overview of robotic liver resection procedures

		Total *n* (%)
Major resections		41 (41.0)
	Right hemihepatectomy	11 (11.0)
	Left hemihepatectomy	10 (10.0)
	Extended right hemihepatectomy	2 (2.0)
	Extended left hemihepatectomy	1 (1.0)
	Resection of 3 segments	12 (12.0)
	Resection of 4 segments	5 (5.0)
Minor resections		59 (59.0)
	Left lateral liver resection	24 (24.0)
	Bisegmentectomy	16 (16.0)
	One segment resection	17 (17.0)
	Atypical one segment resection	2 (2.0)
Total		100 (100.0)

### Learning curve analysis with phases

To analyze the progression of surgical performance in robotic liver resections, we adopted the competency-proficiency-mastery framework proposed by Kuemmerli et al. [16]. This framework provides a standardized approach to defining surgical learning curves. While this model typically includes three phases, we identified four distinct phases in our analysis based on trends in cumulative average operation time, Iwate difficulty scores, and statistical breakpoints: (1) Competency (Cases 1–20), (2) Proficiency (Cases 21–30), (3) Early Mastery (Cases 31–65), and 4) Full Mastery (Cases 66–100). This division allowed us to capture additional nuances in the surgeon’s progression, particularly during the transition to mastering increasingly complex procedures.

Cumulative averages were used to assess trends in operation time and Iwate difficulty scores, which smooth out individual case variability and highlight overall learning progression. For other parameters, such as intraoperative blood loss (IBL) and postoperative morbidity, mean values and frequencies were compared across the four phases using statistical tests, as summarized in Table 4. Notably, 8 (88.9%) out of 9 conversions occurred before the 66th procedure, spanning the Competency, Proficiency, and Early Mastery phases. In the Full Mastery phase (Cases 66–100), only one conversion was observed. This conversion was necessitated by the intraoperative detection of portal vein infiltration, requiring a portal vein resection and reconstruction. For enhanced manual control, the surgeon switched to an open approach following robotic parenchymal dissection to complete the portal vein reconstruction safely and effectively.

**Table 4:** Comparative analysis of outcomes during learning phases

	Phase 1 *n* or mean (% or SD)	Phase 2 *n* or mean (% or SD)	Phase 3 *n* or mean (% or SD)	Phase 4 *n* or mean (% or SD)	*P*-value
Sex					0.605
Male	10 (50.0)	7 (70.0)	20 (57.1)	23 (65.7)	
	10 (50.0)	3 (30.0)	15 (42.9)	12 (34.3)	
Female					
Age; years	56.2 (16.9)	66.9 (8.0)	64.2 (12.5)	61.7 (11.3)	0.312
BMI; kg/m^2^	26.2 (4.1)	27.6 (3.8)	27.4 (5.0)	28.4 (5.8)	0.554
Iwate difficulty score	5.9 (1.4)	9.2 (1.3)	7.9 (2.2)	8.0 (1.9)	< 0.001
Difficulty level					< 0.001
Intermediate (4–6)	16 (80.0)	0 (0.0)	10 (28.6)	9 (25.7)	
Advanced (7–9)	4 (20.0)	5 (50.0)	15 (42.9)	20 (57.1)	
Expert (10–12)	0 (0.0)	5 (50.0)	10 (28.6)	6 (17.1)	
Operation time; min	264.7 (106.6)	450.8 (125.1)	298.0 (97.5)	309.2 (104.4)	0.002
LOS; days	7.4 (3.1)	23.5 (17.4)	11.7 (10.0)	10.5 (8.9)	0.001
IBL; ml	359.5 (344.4)	675.0 (400.9)	336.8 (308.5)	244.7 (203.7)	0.012
Intraoperative blood transfusion	1 (5.0)	6 (60.0)	4 (11.4)	1 (2.9)	< 0.001
Overall morbidity	3 (15.0)	8 (80.0)	8 (22.9)	9 (25.7)	0.001
Liver surgery-related morbidity	1 (5.0)	2 (20.0)	4 (11.4)	5 (14.3)	0.633
Conversion	1 (5.0)	3 (30.0)	4 (11.4)	1 (2.9)	0.054
Previous abdominal surgery	10 (50.0)	8 (80.0)	20 (57.1)	16 (45.7)	0.266
Extent of resection					< 0.001
Major	0 (0.0)	8 (80.0)	15 (42.9)	18 (51.4)	
Minor	20 (100.0)	2 (20.0)	20 (57.1)	17 (48.6)	
Tumor dignity					0.069
Malignant	12 (60.0)	10 (100.0)	27 (77.1)	29 (82.9)	
	8 (40.0)	0 (0.0)	8 (22.9)	6 (17.1)	
Benign					
Size of tumor; cm	65.3 (41.9)	62.8 (27.3)	51.7 (37.1)	50.9 (34.5)	0.279
R status in malignant cases					0.077
R0	12 (100.0)	8 (80.0)	27 (100.0)	27 (93.1)	
R1	0 (0.0)	2 (20.0)	0 (0.0)	2 (6.9)	

*BMI* body mass index, *IBL* intraoperative blood loss, *LOS* length of stay, *SD* standard deviation

**Fig. 1:**
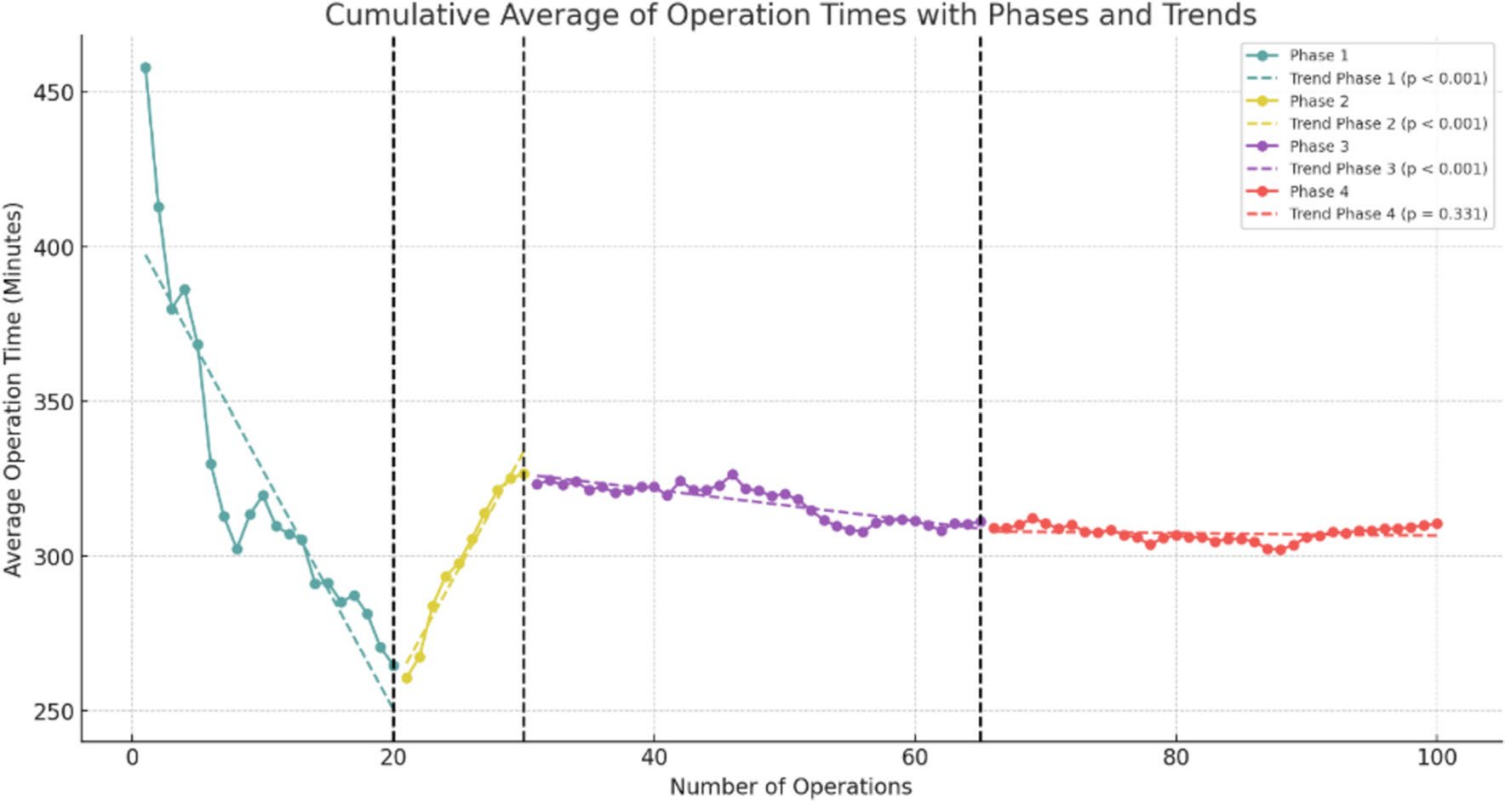
Cumulative average of operation times with phases and trends: cumulative average curves display the overall trends in operation times across all cases

**Fig. 2:**
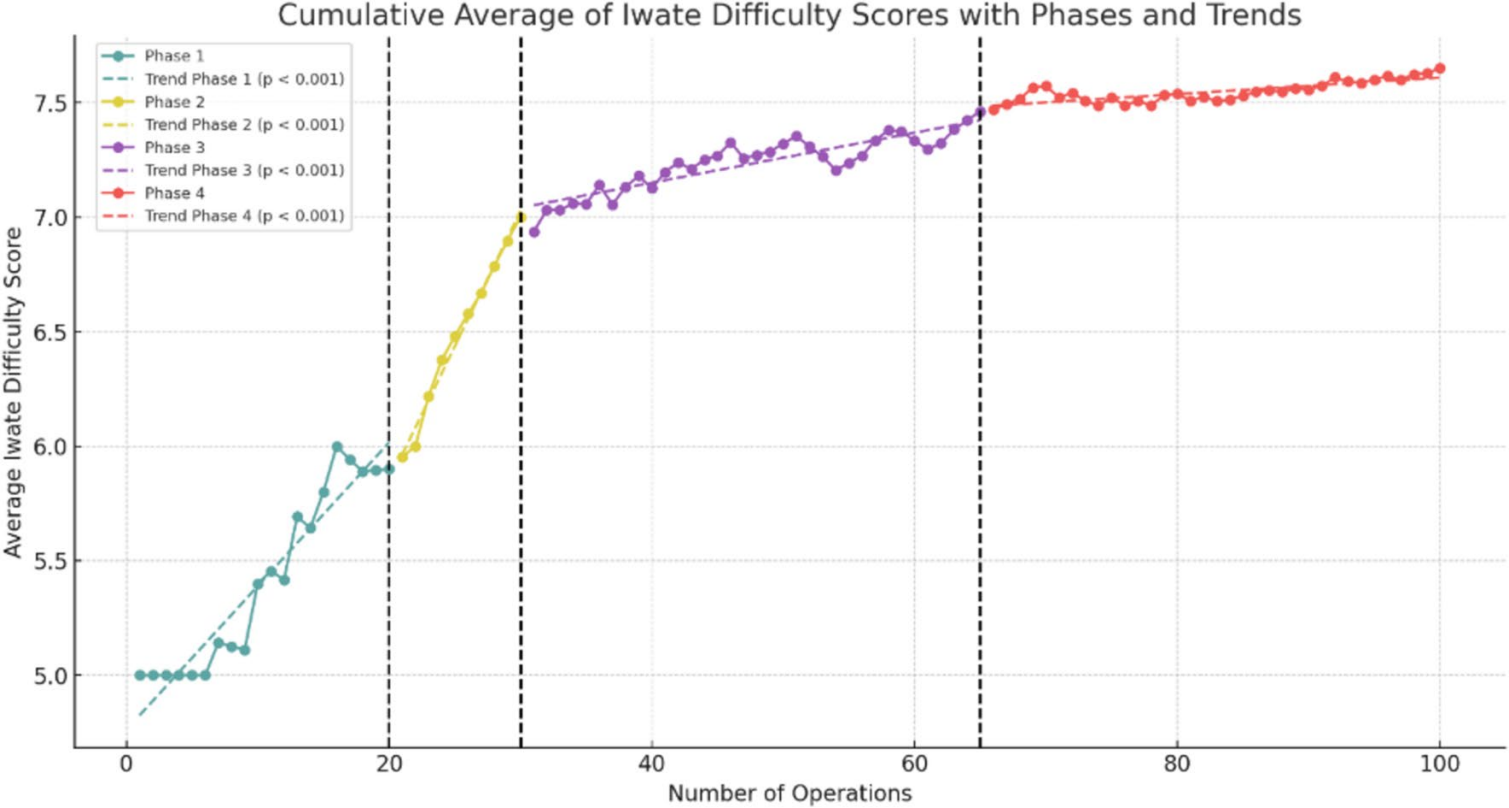
Cumulative average of Iwate difficulty scores with phases and trends: Cumulative average curves illustrate the progression in case complexity across all cases

**Fig. 3:**
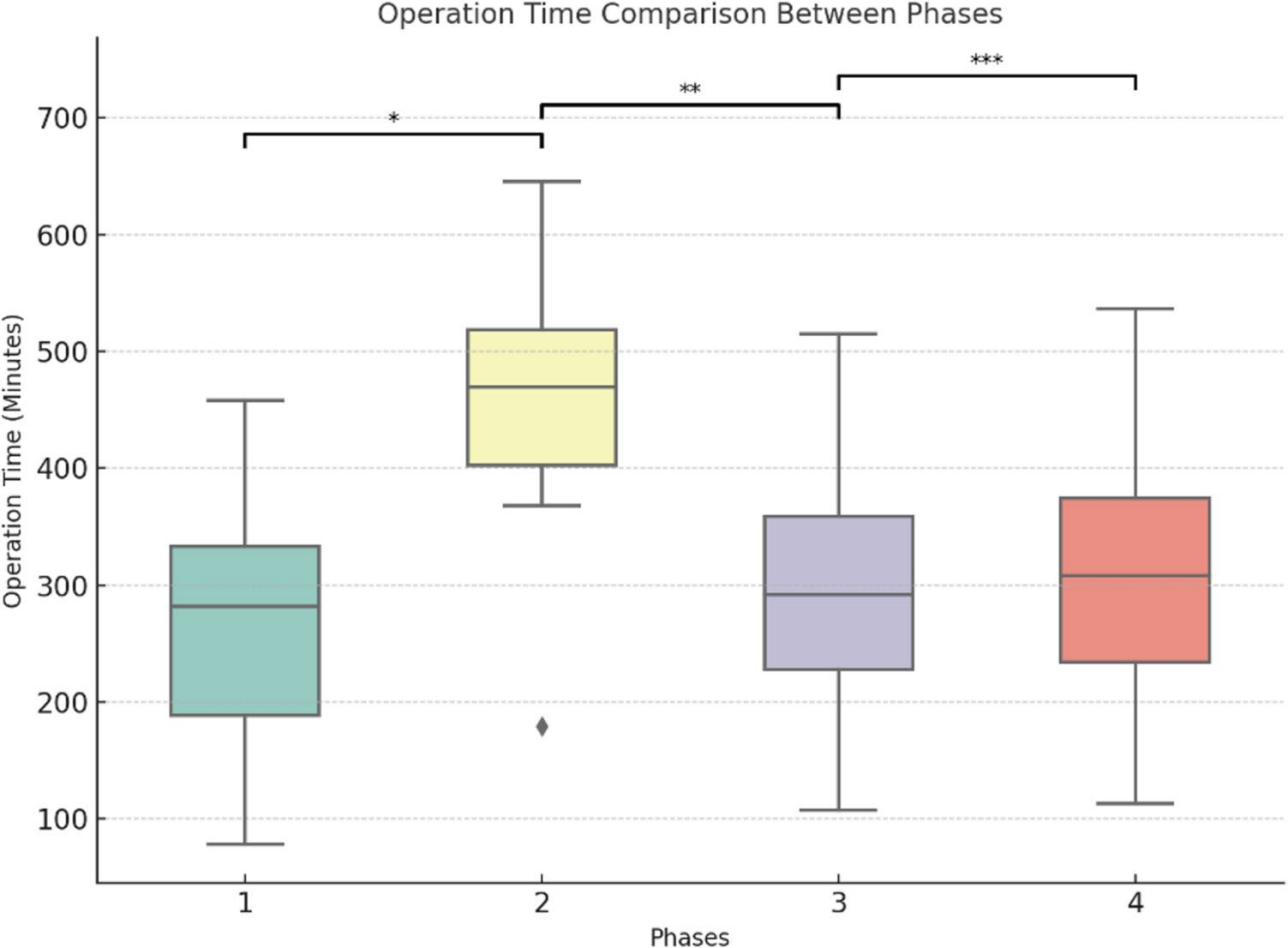
Operation time comparison between phases: box plots represent the distribution of individual operation times within each phase. *Phase 1 vs Phase 2: *p* < 0.001. **Phase 2 vs Phase 3: *p* < 0.001. ***Phase 3 vs Phase 4: *p* = 0.438

**Fig. 4:**
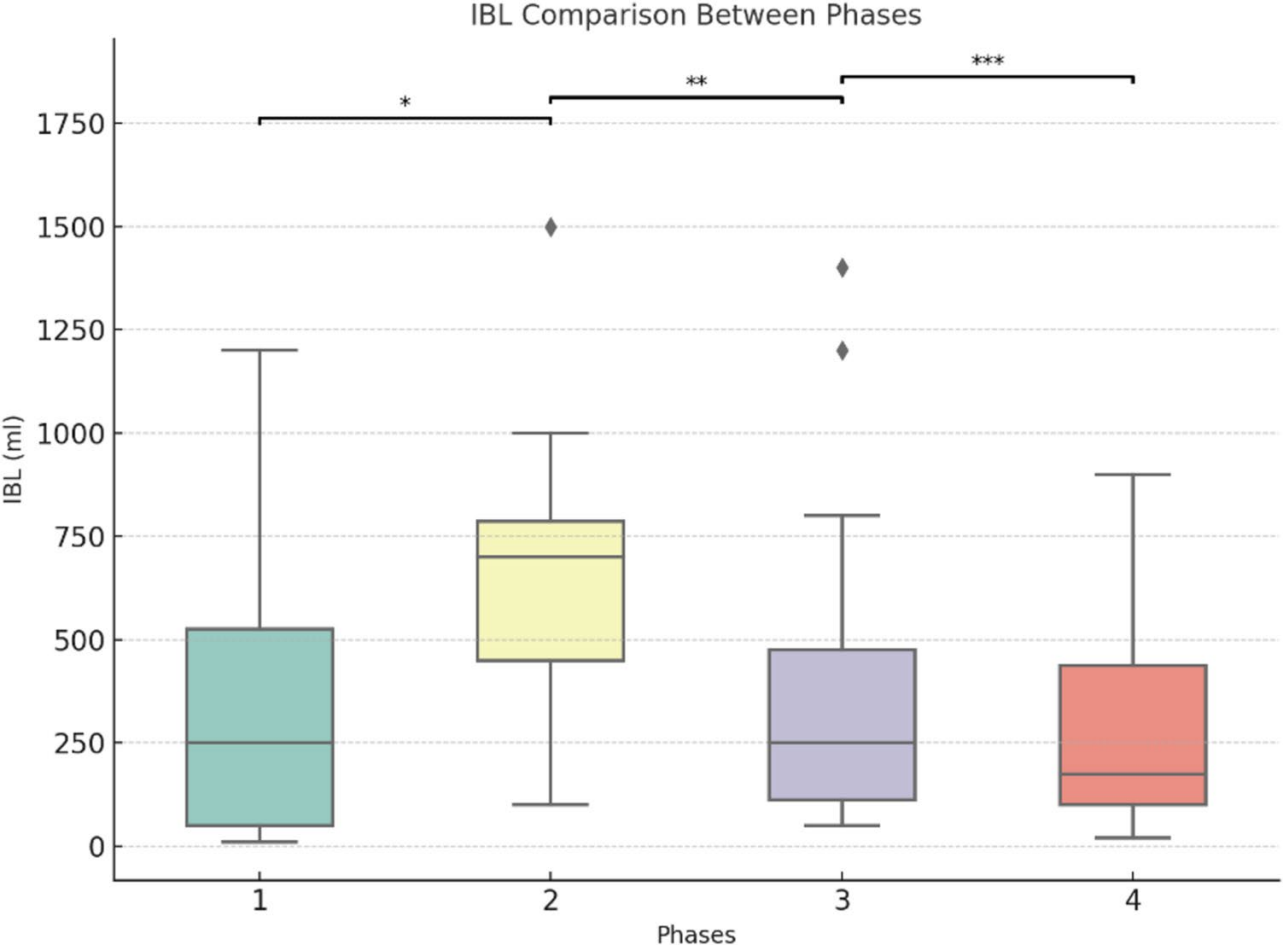
IBL comparison between phases: box plots represent the distribution of intraoperative blood loss (IBL) within each phase. *Phase 1 vs Phase 2: *p* = 0.028. **Phase 2 vs Phase 3: *p* = 0.007. ***Phase 3 vs Phase 4: *p* = 0.228. IBL: intraoperative blood loss

**Fig. 5:**
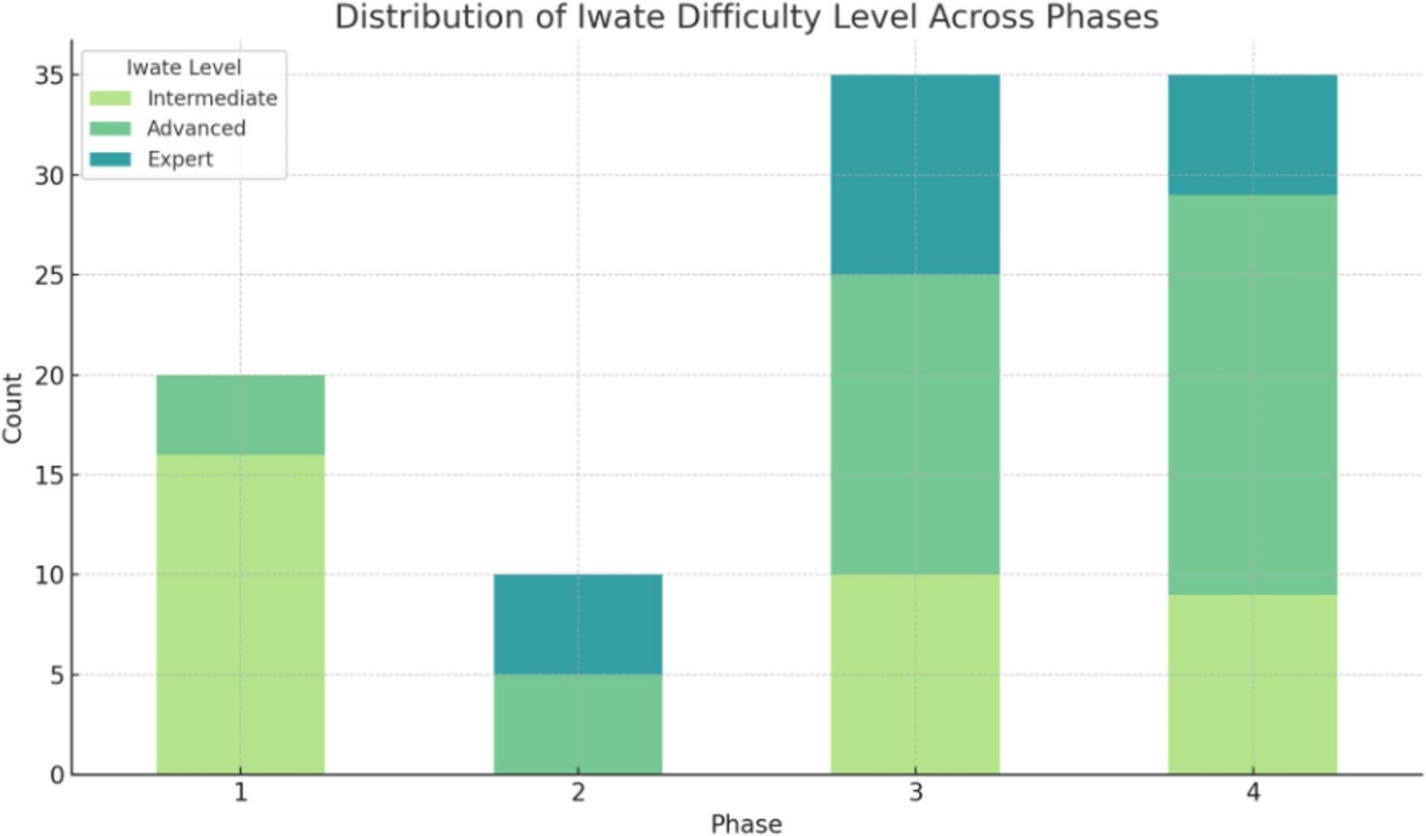
Distribution of Iwate difficulty level across phases: bar chart illustrating the distribution of Intermediate, Advanced, and Expert cases within each phase

The cumulative average curves (Figs. 1 and 2) smooth individual variations, providing an overview of long-term trends, while box plots (Figs. 3 and 4) highlight intra-phase variability, and the bar chart (Fig. 5) illustrates the distribution of case complexity across phases. Tables 5 and 6 provide the corresponding statistical summaries for operation times and Iwate difficulty scores by phase, derived from Figs 1 and 2, respectively.

**Table 5:** Statistical summary of operation times by phase

Phase	Slope	Intercept	R-squared	*P*-value	Interpretation
1	− 7.73	405.13	0.802	< 0.001	Significant decrease in average operation time
2	7.61	105.52	0.966	< 0.001	Significant increase in average operation time
3	− 0.50	341.70	0.756	< 0.001	Moderate decrease in average operation time
4	− 0.04	310.68	0.029	0.331	Insignificant slight change in operation time

**Table 6:** Statistical summary of Iwate difficulty scores by phase

Phase	Slope	Intercept	R-squared	*P*-value	Interpretation
1	0.06	4.76	0.910	< 0.001	Significant increase in difficulty
2	0.12	3.48	0.988	< 0.001	Very strong increase in difficulty
3	0.01	6.71	0.806	< 0.001	Slight increase in difficulty
4	0.00	7.25	0.636	< 0.001	Very slight change in difficulty

Below, we provide a detailed analysis of each phase.

## Phase 1: Competency (Cases 1–20)

In the competency phase, the surgeon focused on acquiring basic robotic skills and adapting to the technical demands of robotic liver surgery. The majority of cases in this phase were minor resections, allowing the surgeon to build confidence and familiarity with the robotic platform.

- **Operation time:** A steep decline in cumulative average operation time (slope = − 7.73, *p* < 0.001) was observed, reflecting rapid improvement in efficiency. This steep learning curve is characteristic of the initial adaptation to a novel surgical technique.
- **Iwate difficulty score:** A moderate increase in the cumulative average score (slope = 0.06, *p* < 0.001) was observed, corresponding to a gradual increase in case complexity as the surgeon’s confidence grew.
- **IBL:** Mean IBL was 359.5 ± 344.4 ml, consistent with simpler cases requiring less hemostatic control.
- **Postoperative outcomes:** Overall morbidity was 15.0%, and liver surgery-related morbidity was rare at 5.0%.

## Phase 2: Proficiency (Cases 21–30)

This phase marks the transition from basic skills to proficiency, with the surgeon beginning to tackle more complex cases, including major resections. This shift required the application of previously acquired skills to more technically demanding procedures.

- **Operation time:** Cumulative average operation time increased temporarily (slope = 7.61, *p* < 0.001), reflecting the added challenges of major resections.
- **Iwate difficulty score:** A sharp rise in the cumulative average score (slope = 0.12, *p* < 0.001) indicated a marked increase in case complexity. This phase represents a pivotal point where the surgeon expanded indications for robotic liver surgery.
- **IBL:** Mean IBL increased significantly (*p* = 0.028) compared to Phase 1, consistent with the higher complexity of cases.
- **Postoperative outcomes:** Morbidity peaked at 80%, reflecting the surgeon’s ongoing adaptation to handling challenging cases.

## Phase 3: Early mastery (Cases 31–65)

The early mastery phase represents a period of stabilization, where the surgeon began to refine techniques and achieve greater consistency in performance. Both minor and major resections were performed with increasing efficiency and control.

- **Operation time:** Cumulative average operation time began to decrease significantly (slope = − 0.50, *p* < 0.001), demonstrating growing efficiency despite case complexity.
- **Iwate difficulty score:** The cumulative average score continued to rise at a slower rate (slope = 0.01, *p *< 0.001), reflecting the surgeon’s ability to handle a balanced mix of advanced procedures.
- **IBL:** Mean IBL decreased significantly (*p* = 0.007) compared to Phase 2, suggesting improved hemostatic control during surgery.
- **Postoperative outcomes:** Overall morbidity declined to 22.9%, indicating better management of complex procedures as skills improved.

## Phase 4: Full mastery (Cases 66–100)

In the full mastery phase, the surgeon demonstrated stable performance with consistent outcomes across increasingly complex cases. This phase signifies the culmination of the learning curve, with the surgeon achieving mastery in robotic liver surgery.

- **Operation time:** Cumulative average operation time stabilized, with no significant changes observed (slope = − 0.04, *p* = 0.331).
- **Iwate difficulty score:** The cumulative average score plateaued (slope = 0.00, *p* < 0.001), reflecting consistent handling of complex cases.
- **IBL:** Blood loss remained stable, with no significant difference compared to Phase 3 (*p* = 0.228).
- **Postoperative outcomes:** Morbidity stabilized at 25.7%, reflecting the surgeon’s consistent ability to manage technically demanding cases with acceptable complication rates.

## Discussion

This study provides key insights into the learning curve for robotic liver surgery, emphasizing the phased progression and specific benchmarks required to achieve proficiency. Based on 100 consecutive robotic liver resections, our findings indicate that approximately 65 procedures are necessary for a surgeon with prior experience in open and laparoscopic liver surgery to attain full mastery. These results are consistent with existing literature, which suggests case numbers between 50 and 60 as thresholds for overcoming the learning curve in minimally invasive liver surgery [12, 13, 26–28].

The structured progression through the four identified phases—Competency, Proficiency, Early Mastery, and Full Mastery—illustrates the systematic development of technical skills and operational efficiency. During the Competency phase (Cases 1–20), significant reductions in operation time were observed as the surgeon became accustomed to the robotic platform and mastered foundational procedures. These findings align with previous studies reporting a steep initial learning curve in robotic liver surgery, which can be mitigated through focused training and experience [26, 29, 30].

In the Proficiency phase (Cases 21–30), the surgeon began performing major resections, resulting in increased operation times and Iwate difficulty scores. This phase represents a critical transition, during which the surgeon expanded their scope to include more technically complex procedures. Similar trends, such as improvements in efficiency and other intra- and postoperative outcomes, have been observed after 30 procedures in other studies [31, 32]. The Early Mastery phase (Cases 31–65) was characterized by a gradual reduction in operation times and increasing case complexity, highlighting the surgeon’s growing consistency and technical refinement. By the Full Mastery phase (Cases 66–100), operation times plateaued, reflecting a high level of proficiency and efficiency. The stable yet elevated Iwate difficulty scores during this phase underscore the surgeon’s ability to manage complex cases effectively and consistently. Alongside these developments, the proportion of major resections and malignant cases increased as the surgeon advanced through the phases, reflecting a growing ability to handle high-complexity procedures with confidence. This trend is further emphasized by the declining conversion rates, with only one conversion occurring in the Full Mastery phase due to a challenging portal vein infiltration, which necessitated a portal vein resection and reconstruction.

These findings are supported by a systematic review of 60 studies, which defined milestones for competency, proficiency, and mastery after 34, 50, and 58 procedures, respectively [16]. In a related context, a single-center study analyzing 500 robotic hepatectomies identified learning curve thresholds of 75 cases for minor resections (*n* = 215), 100 for major resections (*n* = 154), and 57 for technically challenging minor resections (*n* = 131) using cumulative sum (CUSUM) analysis [33]. Such benchmarks contextualize our results and highlight the importance of standardized training protocols and structured mentorship to facilitate surgeons’ progression through the learning curve.

The advantages of robotic platforms, including stable three-dimensional visualization, enhanced dexterity through articulated instruments, and the elimination of physiological tremors, provide distinct benefits over conventional laparoscopy [17, 34, 35]. Despite these advantages, one of the primary criticisms of robotic liver surgery has been the longer operation times compared to laparoscopic approaches [36–38]. Our analysis demonstrates that operation times decreased substantially as the surgeon advanced along the learning curve, ultimately narrowing the performance gap between robotic and laparoscopic techniques.

Notably, the surgeon’s prior experience in open and laparoscopic liver surgery likely shortened the learning curve for robotic liver resections, a trend also observed in other robotic abdominal procedures [39, 40]. The learning curves for laparoscopic liver resections (LLR) and robotic liver resections (RLR) demonstrate distinct yet comparable patterns of progression. In robotic surgery, concomitant training in both RLR and LLR showed a similar learning curve of 40 cases for low and intermediate difficulty procedures, highlighting the potential for parallel training pathways in minimally invasive liver surgery [41]. For complex laparoscopic hepatectomy, significant improvements in operative outcomes, including reduced operation time, blood loss and mortality, were observed after approximately 20 cases, marking a relatively short learning curve for experienced surgeons [42]. Moreover, second-generation laparoscopic surgeons demonstrated a shorter learning curve compared to pioneer surgeons, underscoring that structured fellowship programs can effectively accelerate the learning process and promote the safe adoption of LLR [43]. This underscores the potential value of laparoscopic training as a foundation for robotic surgery, suggesting that comprehensive laparoscopic experience may accelerate the transition to robotic platforms.

In addition to individual skill acquisition, team dynamics play a critical role in robotic liver surgery. The surgeon’s proficiency is closely tied to the collective performance of the surgical team, including the assisting surgeon and operating room staff. Standardized procedures and consistent team composition are essential for facilitating smoother transitions through the learning phases and minimizing variability in surgical outcomes.

While these findings offer valuable insights, the study is limited by its retrospective design and focus on a single surgeon’s experience, which may constrain the generalizability of the results. Future research should include broader cohorts of surgeons with varying levels of experience to validate these observations. Prospective multicentric trials are needed to establish optimal training pathways and refine benchmarks for robotic liver surgery proficiency, thereby contributing to improved surgical outcomes and patient care.

## Conclusion

This study highlights the phased progression of the learning curve in robotic liver surgery, identifying four key phases—Competency, Proficiency, Early Mastery, and Full Mastery. Approximately 65 robotic liver resections are required for surgeons with prior experience in open and laparoscopic techniques to achieve consistent proficiency, enabling them to manage complex cases efficiently.

The observed improvements in operation times and intraoperative parameters underscore the importance of structured training and mentorship. Future multicentric studies are needed to validate these findings and establish evidence-based pathways for achieving mastery in robotic liver surgery. This study reaffirms the potential of robotic platforms to enhance surgical performance, provided surgeons receive comprehensive training and institutional support.
